# Organized Sport Participation and Physical Activity Levels among Adolescents with Functional Limitations

**DOI:** 10.3390/sports5040081

**Published:** 2017-10-19

**Authors:** Kwok Ng, Pauli Rintala, Yeshayahu Hutzler, Sami Kokko, Jorma Tynjälä

**Affiliations:** 1 Faculty of Sport and Health Sciences, University of Jyvaskyla, 40014, Jyvaskyla, Finland; Pauli.rintala@jyu.fi (P.R.); sami.p.kokko@jyu.fi (S.K.); jorma.a.tynjala@jyu.fi (J.T.); 2 Wingate Academic College, Wingate Institute, Netanya 42902, Israel; shayke@wincol.ac.il

**Keywords:** salutogenesis, chronic disease, epilepsy, visual impairment, International Classification of Functioning, Disability and Health (ICF), generalized resistance resources

## Abstract

Sufficient and regular physical activity is considered a protective factor, reducing the onset of secondary disability conditions in adolescents with chronic diseases and functional limitations. The aim of this study was to explore whether participation in organized sport may be associated to higher levels of physical activity in adolescents with functional limitations, based on a national representative sample. Data from the Health Behaviour in School-aged Children (HBSC) study collected in Finland from two data collection rounds (2002 and 2010) were conducted and pooled from adolescents aged between 13 and 15 years old with functional limitations (*n* = 1041). Differences in self-reported physical activity over the past week and participation in organized sport activity were analysed for each function. Overall, four in ten (*n* = 413) participated in organized sport and were significantly (*p* < 0.001) more physically active (mean = 4.92 days, SD = 1.81) than their non-participating (mean = 3.29, SD = 1.86) peers with functional limitations. Despite low population prevalence, adolescents with epilepsy or visual impairments were the least active if they were not participating in organized sport, yet were the most active if they were involved in organized sport. Participating in organized sport appears to be an important factor promoting resources for maintaining recommended levels of physical activity in Finnish adolescents with functional limitations.

## 1. Introduction

Given the important role of physical activity in the prevention of non-communicable diseases [1], studies of adolescents with long-term illnesses or disabilities (LTID) have been gaining much attention. Adolescents with LTID can comprise of non-specified chronic diseases. During adolescence, children experience many biopsychosocial changes that influence the transition into adulthood, making it a critical period for study [2]. The physiological benefits from regular participation in physical activity have been well-documented [3], and so have the psychosocial aspects of health, to a lesser extent [4]. Despite these known benefits, few adolescents with LTID take part in regular physical activities, and the extent of participation varies by different functional limitations. For example, from the Finnish School-aged Physical Activity (SPA) study, adolescent boys with motor impairments reported a mean average of 3.5 days of at least 60 min of moderate to vigorous physical activity per day (MVPA), whereas adolescent boys with visual impairments reported a mean average of 4.8 days of MVPA [5]. According to the World Health Organization, moderate physical activity is considered to be an activity requiring three to six times more energy than sitting quietly (that is, at resting metabolic rate (MET)), and vigorous physical activity requires more than six times relative to this resting MET.

There are known issues that adolescents with LTID face when it comes to the promotion of physical activity, and are noted as barriers and facilitators to physical activity. The barriers to physical activity in adolescents with LTID may include issues such as low self-confidence to perform exercises [6,7,8]; fear of the individual’s health condition impeding on exercising [6,7,9,10], lack of professional knowledge and attitudes of others [7,11,12], as well as physical environmental factors [7,11]. Yet, known facilitators for increasing participation in physical activity have primarily been related to personal and inter-personal factors such as; having fun, involvement with peers; perceived health benefits and own motivation to be active [7,13,14]. 

When exploring the physical activity levels among adolescent groups with different functional limitations, a salutogenic approach [15] was used as a theoretical framework in this study. According to Salutogenesis, a person copes with external or internal stressors by a sense of coherence (SOC) and the utilization of generalized resistance resources (GRRs). The SOC can predict the way a person lives their life despite some health condition. Individuals can deal with their existing conditions by structuring their life into manageable ways and finding meaningfulness in their activities. This is a state of mind that people have, with studies among adolescents demonstrating stronger SOC are related to increased quality of life, reduced substance use, more coping with difficulties [16]. Physical activity was the strongest predictor for SOC among Finnish adolescents [17]. Through physical activities, adolescents constantly face stressors that reinforce the salutogenic approach to health. Physical activities can take place from an array of settings, where organized sports are the most common type among adolescents in the western culture [18]. Participation in organized sports has been reported to have many benefits for society through the promotion of inclusion, health promoting behaviours [14], as well as the individual’s social and mental resources [4]. However, there is a lack of evidence of organized sport as GRRs for adolescents with LTID. The GRRs are either available at the personal or environmental level, and are utilized for enhancing health [15]. 

A standardised framework for describing health and health-related states is the WHO International Classification of Functioning, Disability and Health (ICF) [19]. The main ways of operationalising the ICF are to use the alphanumeric coding system organised in lists. One list is for body structures and body functions. The other list is for indicating actions in life areas as activities and participation. One such code is the engagement in sports (d9201), and several studies point out the barriers to [20] and benefits of full participation in organized sport [21]. Due to favourable social attitudes, accessibility to venues and programs adolescents from the general population context who take part in organized sport activities have opportunities to increase their positive perceptions of physical competence. This combination has been demonstrated to continue into physical activity habits during adulthood [22,23] thus, suggesting organized sport may be an example of effective GRRs. Moreover, organized sport participants with LTID are more likely to meet the international physical activity recommendations for health than non-participants [24].

Less is known about the associations with participation in organized sport and physical activity outcomes among adolescents with LTID [25]. This is even more evident in studies that make comparisons among adolescents with functional impairment. The resources needed to facilitate regular physical activity for one type of functional limitation, such as breathing difficulties are likely to differ from, for example, visual impairments. Therefore, the purpose of this study was to explore whether participation in organized sport may be linked to increased physical activity and therefore considered as a GRR in adolescents with functional limitations, based on a national representative sample.

## 2. Materials and Methods

### 2.1. Participants

Data extracted from the Finnish version of the WHO cross-national collaborative Health Behaviour in School-aged Children (HBSC) study were used. The HBSC study is a quadrennial cross-sectional study that involves adolescents aged between 11 and 15 years old. Minority populations such as adolescents with long-term illnesses or disabilities (LTID) in one data collection cycle in 2002 were pooled with another cycle in 2010 to improve the statistical power. On both occasions, the data was collected in the Spring months of the year. 

The sampling procedures were identical in both data collection years. This involved producing a sample frame based on the total number of pupils supplied through the national school register. From this information, a cluster sampling method that took into account the number of pupils in the school, and from inside the selected school, the class was chosen at random. National representativeness was based at two levels, one at the regional level, and the second, based on municipality types; urban, semi-urban, and rural. Adolescents in Swedish speaking schools, special educational schools, or home schooled were not included. 

For this study, respondents from the adolescents in 13 (M_age_ = 13.7 y, SD = 0.30) and 15 (M_age_ = 15.7 y, SD = 0.32) year old age groups were used because they were given the essential questions used in this analysis. The survey was administered by teachers in school classrooms during a lesson. Teachers were given instructions on how to provide assistance if necessary, thus ensuring standardise protocols throughout the data collection. The starting sample size from the HBSC study in 2002 (*n* = 3932) and 2010 (*n* = 4874) were pooled and cleaned to filter out unreadable data, non-respondents, outliers in age range, removal of missing values to the final sample, and pupils who did not have LTID (*n* = 1041). Details of these procedures can be found elsewhere [26]. This study adds to previous analyses [27] from the same dataset by focusing on participants and non-participants of organized sport after taking into account different functional limitation categories. The survey was carried out voluntarily, through passive consent, and the respondent had the right to withdraw. No personal identifiers were used, and data was anonymous. The Finnish HBSC study was approved by the Finnish Teachers Trade Union and the Finnish National Agency for Education when the survey was collected the first time. Approval has not been requested in each survey year, and no known objections have been raised. There were no incentives or rewards for participation in the survey. Requests to access the data can be made through the international HBSC data bank in Bergen, Norway—http://www.hbsc.org. 

### 2.2. Materials

Adolescents were asked to respond to the following question, “Do you have a long-term illness, disability, or medical condition (like diabetes, arthritis, allergy, or cerebral palsy) that has been diagnosed by a doctor? Please do not include learning disabilities”. Response options were “yes” and “no”. A further question was available to categorise functional limitations to adolescents who selected “yes”. This question was only available in the 2002 and 2010 questionnaires, and was worded as; “If you answered yes, does this disability, illness, or medical condition cause you (1) difficulty seeing things (does not include prescription eye glasses), (2) difficulty in hearing what others say, (3) difficulty in speaking to others, (4) difficulty in moving around, (5) difficulty in handling objects, (6) difficulty in breathing, or (7) epileptic seizures (fits)?” The respondents proceeded to check boxes under columns of “yes” or “no” for each functional limitation. This question was missing from 2006 and 2014 survey, and pooled data was only from the 2002 and 2010 surveys. For the study analyses, respondents who checked “difficulty in speaking to others” (*n* = 7), “difficulty in hearing what others say” (*n* = 21), or both speaking and hearing difficulties (*n* = 1) were combined into a communication impairment category. In addition, a motor impairment category was composed of the combination of “difficulty in moving around” (*n* = 50), “difficulty in handling objects” (*n* = 11), and both moving and handling object difficulties (*n* = 6). Respondents who reported “yes” to the screener question about LTID, but did not check any functional limitation boxes were grouped into a “not specified” category. The “not specified” category is an important group to indicate the variety of other existing health conditions such as chronic diseases and rare conditions. The International Classification of Diseases 10th revision has over 14,000 codes for various conditions, and there are too many to list in a child-friendly format in national representative survey. Co-existing functional limitations of more than two categories did exist (*n* = 11) and were removed from the analysis. 

Physical activity was measured through a single self-reported item in both surveys. A preamble to contextualise moderate intensity physical activity was presented before the following question,

“Over the past 7 days, on how many days were you physically active for a total of at least 60 min per day? Please **add up** all the time you spent in physical activity each day.”

*(emphasis is the same as in the questionnaire)*

The response options ranged from 0 days through to 7 days, with a checkbox on each whole number increment between and including this range. This single item has been used in the HBSC study since 2001 and has featured as a key indicator of physical activity to meet the WHO physical activity recommendations [28] within international surveys [25]. Moderate correlation coefficients (r = 0.46; *p* < 0.001) with objective measures have been reported [29] and the item test-retest scores among a representative sample of Finnish adolescents in general schools have indicated acceptable reliability with ICC values ranged between 0.7 and 0.8, depending upon age and gender [30]. 

Groups of non-participants and participants of organized sport were based on a single item sports club membership question. The question was, “Are you a member of a sports club?” There were three response options, “No”, “Yes, and I am training in a sports club”, and “Yes, but I do not participate in training”. The main purpose of the study was aligned with the framework of the ICF, and members of a sports club, but did not participate in training (*n* = 40), were grouped with the group of non-participants. Confirmed through Kruskal-Wallis pairwise comparisons between the three groups, the number of physically active days between the “no” and “yes, but I do not participate in training” groups did not differ (*p* = 0.172). Whereas, there were significant differences between the group of “yes, and I am training in a sports club” with the other two groups (*p* < 0.001). 

### 2.3. Analyses

Potential confounders that could influence the levels of physical activity were explored, including gender, age [31], and year of data collection [24]. The confounders were used in adjusting the models to describe the differences of physical activity levels between non-participant and participants of organized sport. To check for confounding on the dependent variable, independent T-tests were used for gender, age, and year of data collection. 

To report the adjusted mean levels of physical activity by gender, age, and year of data collection, a univariate analysis of covariance test [32] were performed with no independent variables, and repeated for each functional limitation category. Statistical analyses were conducted through non-parametric *t*-test (Mann-Whitney) using SPSS 24.0 between organized sport participants and non-participants among the various functional impairment categories. Statistical significance was tested at *p* < 0.05. Cohen’s d effect sizes were computed and reported. 

## 3. Results

Approximately four in 10 (*n* = 413/1041) of the sample participated in organized sport. The sample consisted of slightly more girls than boys (*n* = 591/1041) although there were no differences in organized sports participation (*p* = 0.533). Age groups were evenly distributed, although significantly (χ^2^ = 23.77, *p* < 0.001) more 13 year olds (58.6%) were active participants of organized sports than 15 year olds (41.4%). There were more adolescents from the 2002 data collection (*n* = 561/1041) than in 2010. There was a higher (χ^2^ = 4.02, *p* = 0.045) proportion of participation in organized sports in 2010 (50.1%) than 2002 (43.8%). These distributions varied by LTID category and are reported in Table 1. The most commonly identified LTID category in general school was breathing difficulties (31.1%), and the least was epilepsy (1.9%). Differences in proportion of each functional limitations group, were similar in both 2002 and 2010, except adolescents with motor difficulties in 2002 (*n* = 48) and in 2010 (*n* = 19). The proportion of adolescents with breathing difficulties who were participants of organized sports in 2002 (45.3%) and 2010 (54.7%) were significantly different (χ^2^ = 4.18 *p* = 0.042), whereas other functional difficulty groups involved in organized sports did not differ between the two data collection cycles. 

Mean physical activity levels differed by gender (Girls m = 3.67 SD = 1.94, Boys m = 4.17 SD = 2.07, *t*-test *p* = 0.001), age (13 y m = 4.26 SD = 2.02, 15 y m = 3.63 SD = 1.95, *t*-test *p* < 0.001), and year of data collection (2002 m = 3.54 SD = 1.96, 2010 m = 4.40 SD = 1.97, *t*-test *p* < 0.001). The overall mean PA levels of adolescents with LTID was 3.94 days with a pooled (by year) standard deviation of 1.96. The levels of physical activity between participants of organized sport was significantly higher than of non-participant in the following functional limitation categories; Not specified, breathing, motor, visual, and epilepsy categories (Table 2). The effect size calculations, according to Cohen [33] were large for the categories with except the combination of motor & breathing with a medium sized effect. 

Participating boys in organized sport reported significantly more (*p* = 0.001) MVPA (mean = 5.44 days, SD = 1.49) than girls (mean = 4.59 days, SD = 1.88), whereas there was no significant gender difference among non-participants. There were no significant age differences among organized sport participants, although younger non-participants (13 year old; mean = 4.26 days, SD = 2.02) reported significantly more (*p* = 0.045) MVPA than older (15 year old; mean = 3.62 days, SD = 1.95). 

**Figure 1:**
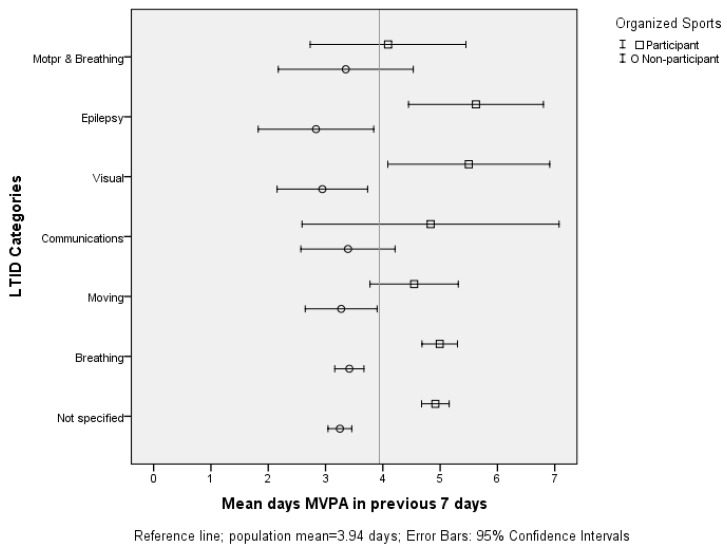
Non-adjusted means and error bars of MVPA by sport club participation.

For each LTID category, participants in organized sport reported more days of 60 min MVPA than what non-participants in their respective category did, in particular adolescents with not specified, breathing, visual and epilepsy groups (Figure 1). Of non-participants, adolescents with breathing difficulties (mean = 3.42 days, SD = 1.81), communicating difficulties (mean = 3.39 days, SD = 1.90), and motor and breathing difficulties (mean = 3.35 days, SD = 2.29) reported, on average, the highest amounts of non-adjusted MVPA. At the other end of the spectrum, non-participant adolescents with visual impairments (mean = 2.94 days, SD = 1.56) and epilepsy (mean = 2.83 days, SD = 1.59) reported, on average, the lowest amounts of non-adjusted MVPA. 

Participants in organized sport with epilepsy were, on average, 2.8 days more active than non-participants with epilepsy. Similar results were noticed with adolescents with reported visual impairments, because participants in organized sport were, on average, 2.6 days more active than their matched non-participant peers. The mean number of days were higher than other functional difficulty groups including difficulties in breathing (mean = 4.99 days, SD = 1.77) and communication (mean = 4.83 days, SD = 2.14).

## 4. Discussion

According to the results of this study, adolescents with LTID reported, on average, more days of physical activity when they were participants of organized sport than their matched non-participant peers. More specifically, the difference between participants and non-participants of organized sport was highest among adolescents with visual impairments or epilepsy. A large effect was observed in the difference of physical activity levels between participants and non-participants of organized sport. The group with the most adolescents with LTID and specified functional limitation was the group of breathing difficulties. 

Consistent with the principle of participating adolescents in organized sport are more physically active than their non-participating counterparts [34], in this study, the levels of physical activity were greater from participants of organized sport than non-participants. These findings compliment what Geidner and Jerlinder [14] recently reported, whereby one of the main reasons for including adolescents with LTID into organized sports is to promote physical activity. The difference in physical activity levels were exemplified among adolescents with visual impairments or epilepsy. On average, more than two days difference in moderate to vigorous physical activity was observed between organized sport participants and non-participants. Adolescents with visual impairments or epilepsy who were non-participants in organized sports had the lowest levels of PA. Considering 30% of adolescents with visual impairments were organized sport participants, these findings concur with previous studies focused on adolescents with visual impairments, where low levels of physical activity were reported [35]. The enhancing benefits of organized sport may explain how Aslan and colleagues [36] reported low amounts of low intensity physical activity during the week, whereas during the weekend, there were increased moderate intensity levels among adolescents with visual impairments. This could be because the majority of adolescent organized sports may have competitions held on weekends in addition to their regular training schedules during the week. Based on this interaction, it may be suggested that participation in organized sports could be seen as a GRR for maintaining physical activity participation and ultimately health benefits. 

Adolescents with LTID may find their health conditions impede their participation in organized sports [10], whereas similar adolescents with desire to be active and find enjoyment from exercise may find they can thrive is such situations [7]. Enrichment through participation of adolescents with LTID in organized sport may also depend on the activities available, accessibility to the sports, the purpose of the club activities, the personnel that run and operate the activities, and the level of inclusion [14]. These determinants act as GRRs in a salutogenic approach to health. With regard to participants with visual impairments, Lieberman and colleagues [37] suggest practitioners should implement successful modifications through awareness of individual needs. As the majority of organized sports are run by volunteers, these dedicated resources are essential in the promotion of physical activities.

Adolescents with epilepsy need to learn to deal with self-management skills, including participation in physical activity and sport [38]. It is especially important, because the risk of injuries during sport is higher than peers without epilepsy [39]. Managing this risk can be mitigated through education and support. The organized sport environment may be perceived to be safer for adolescents with epilepsy and could explain the large differences in physical activity reported in our study. Other determinants that influence participation in leisure activities such as physical activity include age, gender, degree of activity limitations, family preferences and coping, motivation, and environmental resources and supports [6,40]. Additional research is needed to better establish physical activity determinants related to organized sport participation in children with functional limitations. Examples for potential determinants for increased participation that have been indicated in other populations are self-efficacy [8], group cohesion [41], and athletic self-identity [42]. 

The resources available to adolescents with LTID need to be carefully designed. It is often assumed that adolescents with LTID have to cope with stressors in their lives such as maintenance of the condition, sometimes through medication, regular health care visits, poorer mental health, and difficulties to make transitions in life [43]. Therefore, the need to focus on the resources available to the individual and not only the aetiology and treatment are essential ways to improve health. Many studies in the phenomenon of drop-out of physical activity have been attributed to low motivation, bad experiences, a lack of time, and unsuitable activities and there could be further barriers for individuals with disabilities [10]. Instructors with specialist knowledge of inclusion may encourage more engagement in organized sport, as activities may foster more social interaction among participants, and this can lead to increased levels of support between adolescents with and without limitations [44]. Moreover, positive and negative health behaviours are known within organized sport participation, such as reducing substance use, bullying, fighting, and improving sleep [45,46] and require further educating to create a more effective resource for health. 

The study has come from a national representative sample of school-aged children, and we provided an overall picture of the prevalence of functional limitations, physical activity levels, and participation in organized sport among Finnish adolescents. Comparisons among groups were possible because measures were the same allowing the possibility to be combined with larger national and international data sets. However, with large size and self-reporting surveys, it is worthy to note some study limitations. The data was cross-sectional, and no causal inference can be made between participation in organized sport and physical activity levels. Adolescents self-reported their functional limitations and there may be other reporting biases in the exposure and outcome variables. The level of representative data is of the national population, not necessarily of particular impairments, and this should be taken into consideration when interpreting the results. Finally, interpretation of the results are restricted to the general school population and not from participants outside of the HBSC study, such as from special schools and non-Finnish language schools.

## 5. Conclusions

Participation in organized sports is clearly associated with increased levels of physical activity among adolescents with LTID, although the level of association varied by functional limitation. The prevalence of organized sport participation also varied by functional limitation and needs to be addressed in future programs that include adolescents with LTID. Participation in organized sports has the potential to act as GRRs, with a large effect for adolescents with breathing difficulties, visual impairments, and epilepsy. Studies that can help underpin the reasons for the low frequency of participation in organized sports among adolescents with visual or communication impairments are needed. In addition, future studies may need to examine the relationships with other physical activity related behaviours, such as sleep, sedentariness, nutrition, peer and family support, and quality of life. One of the main findings of the current study were in relation to the largest difference in physical activity levels among adolescents with visual impairments, yet only 30% reported participating. More programs that can increase participation in organized sport may enhance the overall physical activity levels in these functional limitation categories.

## Figures and Tables

**Table 1:** Study characteristics by long-term illnesses or disability categories in percentages (column).

	Not Specified	Breathing	Motor	Communication	Visual	Epilepsy	Motor and Breathing
	(*n* = 548)	(*n* = 323)	(*n* = 67)	(*n* = 29)	(*n* = 26)	(*n* = 20)	(*n* = 28)
Gender							
Male	52.2%	60.4%	58.2%	69.0%	42.3%	60.0%	85.7%
Female	47.8%	39.6%	41.8%	31.0%	57.7%	40.0%	14.3%
Age Group							
13	49.8%	45.8%	50.7%	55.2%	42.3%	75.0%	57.1%
15	50.2%	54.2%	49.3%	44.8%	57.7%	25.0%	42.9%
Year of Data Collection							
2002	53.8%	52.3%	71.6%	55.2%	50.0%	40.0%	35.7%
2010	46.2%	47.7%	28.4%	44.8%	50.0%	60.0%	64.3%
Organized Sport							
Non-Participant	58.2%	60.4%	65.7%	79.3%	69.2%	60.0%	60.7%
Participant	41.8%	39.6%	34.3%	20.7%	30.8%	40.0%	39.3%

**Table 2:** Differences in means of moderate to vigorous physical activity per day (MVPA), after adjusting for gender, age, and year of data collection, between participant and non-participant groups.

	Non-Participant (*n* = 628)			Participant (*n* = 413)			MWtest ^a^	Cohen’s
	Mean	LCI	UCI	Mean	LCI	UCI	*p*	d
Not Specified	3.31	3.11	3.51	4.84	4.60	5.07	<0.001	0.90
Breathing	3.80	3.23	3.72	4.90	4.59	5.20	<0.001	0.88
Motor	3.27	2.70	3.84	4.56	3.75	5.37	0.010	0.65
Communication	3.31	2.44	4.17	5.16	3.41	6.91	0.135	0.74
Visual	2.99	2.16	3.82	5.40	4.09	6.70	0.002	1.58
Epilepsy	2.87	1.85	3.88	5.58	4.33	6.82	0.002	1.84
Motor & Breathing	3.66	2.64	4.69	3.61	2.32	4.91	0.351	0.33
Total	3.34	3.20	3.48	4.85	4.67	5.02	<0.001	0.89

^a^ Mann-Whitney U Exact Significant Test (2-tailed). LCI—Lower confidence interval, UCI—Upper confidence interval.
